# Exploration of the Potential Suitable Distribution Range of the *Lyophyllum decastes* Complex in High-Altitude Cold Regions of Southwest China

**DOI:** 10.3390/jof12080571

**Published:** 2026-08-01

**Authors:** Shuwei Wei, Yue Mi, Xiaozhuo Zhang, Bao Qi, Wanyi Wang, Qi Wang, Dongfang Shi, Yu Li

**Affiliations:** 1 Institute of Innovation Science and Technology, Changchun Normal University, Changchun 130032, China; 13630588572@163.com (S.W.); 186097730680@163.com (Y.M.); 2 Key Laboratory of Applied Statistics of Ministry of Education, School of Mathematics and Statistics, Northeast Normal University, Changchun 130024, China; zhangxz722@nenu.edu.cn; 3 Engineering Research Center of Chinese Ministry of Education for Edible and Medicinal Fungi, Jilin Agricultural University, Changchun 130118, China; qibao3712@163.com (B.Q.); wanyiwang369@gmail.com (W.W.); q_wang2006@126.com (Q.W.)

**Keywords:** distribution, *Lyophyllum*, macrofungi, MaxEnt, species diversity

## Abstract

Climate change may alter the abiotic conditions associated with macrofungal occurrence, but projections for taxonomically unresolved species complexes require cautious interpretation. Here, we used an AICc-optimized MaxEnt model to characterize the climate-defined aggregate suitability envelope of records assigned to the *Lyophyllum decastes* (Fr.) Singer complex in Sichuan, Yunnan, and Xizang, Southwest China, under current conditions and four RCP scenarios for the 2050s and 2070s. After spatial thinning, 70 occurrence records compiled from field surveys and published sources were modeled with correlation-filtered bioclimatic variables. The optimized model showed high discrimination for the pooled occurrence dataset (mean AUC > 0.95), and mean temperature of the coldest quarter had the largest contribution (50.8%). Projected changes differed between scenarios, with increasing fragmentation and reductions in highly suitable climate space under several futures. These outputs do not represent a lineage-resolved species distribution, as the occurrence records were not uniformly verified by molecular barcodes, and the model omits host availability, vegetation, soil, land use, dispersal, and other biotic processes. We therefore interpret the maps as a hypothesis-generating, aggregate climatic envelope for the complex that can guide stratified field surveys, molecular sampling, and habitat monitoring rather than as direct evidence of taxon-specific physiological tolerances or realized distributions.

## 1. Introduction

Global warming has become a focal point of public concern. Since the 1970s, there has been growing awareness that global climate anomalies are intricately linked to the interactions between oceans and the atmosphere [1]. Today, the scientific community widely acknowledges that carbon emissions are one of the primary drivers of global warming, with the excessive release of greenhouse gases leading to a rise in global temperatures and increasingly harsh climatic conditions. Climate change exerts a significant impact on species distribution and ecological migration, accelerating the rate of species extinction or proliferation, and is considered one of the greatest threats to global biodiversity in the 21st century, posing a substantial challenge to the conservation of biodiversity in the future [2]. Consequently, utilizing ecological niche models to predict changes in species’ suitable distributions under climate change has become a hot topic in the field of biological conservation. In Earth’s ecosystems, species typically respond to climate change through extinction, adaptation, and migration [2,3]. The migration of many species is markedly positively correlated with temperature fluctuations [4,5], with species gradually shifting towards higher latitudes and altitudes as their habitats migrate [6], leading to a reduction in species diversity in low-latitude, low-altitude regions and intensifying competition among species in high-latitude areas, thereby inducing changes in the structure of natural ecosystems [7]. Research by Li et al. [3], under the scenario of climate change in China, has shown that the suitable distribution area for *Cunninghamia lanceolata* (Lamb.) Hook. has changed, with a northward shift in its centroid, and that precipitation variables have a more significant impact on this species than temperature variables, indicating that the future suitable distribution area will be more fragmented [3]. Furthermore, the selection of different carbon emission scenarios also affects the future distribution of the studied species [8]. Climate-change-induced species invasions are increasing globally, making the effective and sustained management of biodiversity and ecosystems particularly crucial, and it is imperative to consider the impact of climate change on species habitats; thus, species distribution models have become an integral component of conservation plans [9].

Climate change exerts an influence on the distribution characteristics of fungi, potentially altering species diversity across different regions and prompting taxa that would have evolved independently to change their migration patterns [10]. The rise in global temperatures, coupled with human activities and exacerbated climate change, has had a significant impact on the richness and geographic distribution of fungal species [11]. Habitat degradation or loss due to climate change also affects the abundance and spatial distribution of these species [12,13]. As the global average temperature continues to rise, precipitation patterns, relative humidity, and other related environmental factors will undergo changes, and gathering this information will play a beneficial role in understanding the geographical distribution, environmental adaptability, and diversity changes in fungi [14,15,16]. Considering climate change, identifying suitable habitats for fungi could greatly influence their reproduction and survival. Protecting the diversity of fungal species resources; exploring new germplasm resources; and predicting the impact of climate change on species diversity, ecological and geographical distribution, and richness can provide a theoretical basis for the sustainable conservation and utilization of fungi. The climate can directly affect the distribution of species distribution, ecosystems, and landscapes, as well as crop productivity and many other aspects [17]. Early researchers had already discovered that different climatic zones possess their own ecological characteristics, but due to the limitations of the scientific environment at the time, descriptions were based solely on observations of instantaneous climate changes [18,19,20,21]. With the advancement of technology, researchers have gradually constructed various climate models to predict urban ecosystems, large-scale terrestrial ecosystems, or marine ecosystems. The maximum entropy (MaxEnt) model, originating from statistical mechanics, is a universal method for making predictions based on incomplete information, and has been applied in multiple fields [22,23,24,25]. As one of the species distribution modeling methods, MaxEnt estimates the target probability distribution by finding the probability distribution with maximum entropy [23,26,27].

In botanical research, Yi et al. [28] used the MaxEnt model to predict the potential distribution of *Homonoia riparia* Lour., a riparian medicinal shrub, and identified mean annual temperature, elevation, and precipitation during the coldest quarter as important determinants of its suitable habitat. Cao et al. [29] projected that the suitable habitat of *Hylomecon japonica* (Thunb.) Prantl, a perennial herbaceous plant, would expand substantially under several future climate scenarios. Shi et al. [27] employed a parameter-optimized MaxEnt model to simulate the potential distribution of *Litsea cubeba* (Lour.) Pers., an aromatic deciduous tree or shrub. Their results showed that *L. cubeba* was primarily distributed in areas south of the Yangtze River, with a total suitable area of approximately 2.08 × 10^6^ km^2^ and highly suitable areas concentrated mainly in South and East China. In animal research, Mao et al. [30] predicted the potential distribution of *Wasmannia auropunctata*, an invasive ant commonly known as the little fire ant, in China. Their results indicated that this species has considerable potential to establish itself in many areas south of the Yangtze River, and that temperature is an important variable associated with its modeled distribution. Fu et al. [31] modeled the potential distribution of *Platalea leucorodia* Linnaeus, a large wetland waterbird commonly known as the Eurasian spoonbill, and identified elevation, proximity to water, mean temperature of the driest quarter, and precipitation as important environmental factors. He et al. [32] evaluated habitat suitability for the Asian elephant, a large terrestrial mammal, in Xishuangbanna, China, under current climatic conditions. The predicted distribution was compared with available movement trajectories, and elevation, temperature, and precipitation were identified as major variables associated with habitat suitability.

Fungal species distribution modeling has developed considerably in recent years and has been applied to diverse ecological and taxonomic groups across different regions of the world. Previous studies have used species distribution models to investigate the geographic patterns of potentially phytopathogenic fungi at broad spatial scales [10], assess the responses of ectomycorrhizal fungal diversity to climate change in association with host forests [33], and predict the current and future distributions of edible and medicinal macrofungi, including *Ophiocordyceps sinensis* (Berk.) G.H. Sung, J.M. Sung, Hywel-Jones & Spatafora; *Tricholoma matsutake*; *Polyporus umbellatus* (Pers.) Fr.; and *Morchella* spp. [34,35,36,37]. Distribution modeling has also been applied to wood-decaying fungi to evaluate how temperature and precipitation may influence their potential persistence under future climatic conditions [38]. Collectively, these studies demonstrate that species distribution models are increasingly used in fungal biogeography and climate change research.

However, fungal distributions cannot be interpreted solely from climatic predictors. Temperature and precipitation may characterize broad-scale climatic suitability, whereas realized fungal distributions may additionally depend on nutritional strategy, host-plant availability, vegetation type, soil properties, substrate availability, dispersal limitation, land-use change, and human disturbance [33,38,39,40]. These ecological constraints are particularly important when modeling a taxonomically unresolved species complex whose members may differ in trophic strategy and environmental requirements. Therefore, the present study builds upon the existing fungal distribution-modeling literature by using an optimized MaxEnt model to characterize the aggregate climatic envelope of occurrence records assigned to the *Lyophyllum* decastes complex in Sichuan, Yunnan, and Xizang. Instead of assuming an absence of previous macrofungal modeling studies, we focus on the regional and taxonomic uncertainty associated with this complex and evaluate how its pooled climatic envelope may change under future climate scenarios.

The *Lyophyllum decastes* (Fr.) Singer complex comprises closely related and morphologically similar lyophylloid agaric fungi, whose delimitation is complicated by phenotypic plasticity and incomplete molecular verification across historical records [41,42]. Reported members occur in forest, woodland, grassland, and disturbed habitats, and they are valued as edible macrofungi. Importantly, the complex should not be treated as ecologically homogeneous, as some records may represent saprotrophic members whose occurrence depends strongly on substrate and moisture, while *Lyophyllum shimeji* has been experimentally shown to form ectomycorrhizae with conifer seedlings [43]. Mycorrhizal members may therefore track host availability and host migration, while saprotrophic members may respond more directly to litter, woody debris, soil organic matter, and microclimates. Modeling the pooled complex under climate change is useful as a preliminary survey-prioritization exercise, but it cannot resolve lineage-specific niches. Accordingly, our objective is to estimate the aggregate climatic envelope associated with records assigned to the *L. decastes* complex, evaluate how that envelope changes across climate scenarios, and identify areas where field surveys, host assessment, and molecularly resolved sampling should be prioritized.

## 2. Materials and Methods

### 2.1. Occurrence Record Compilation

**Figure 1:**
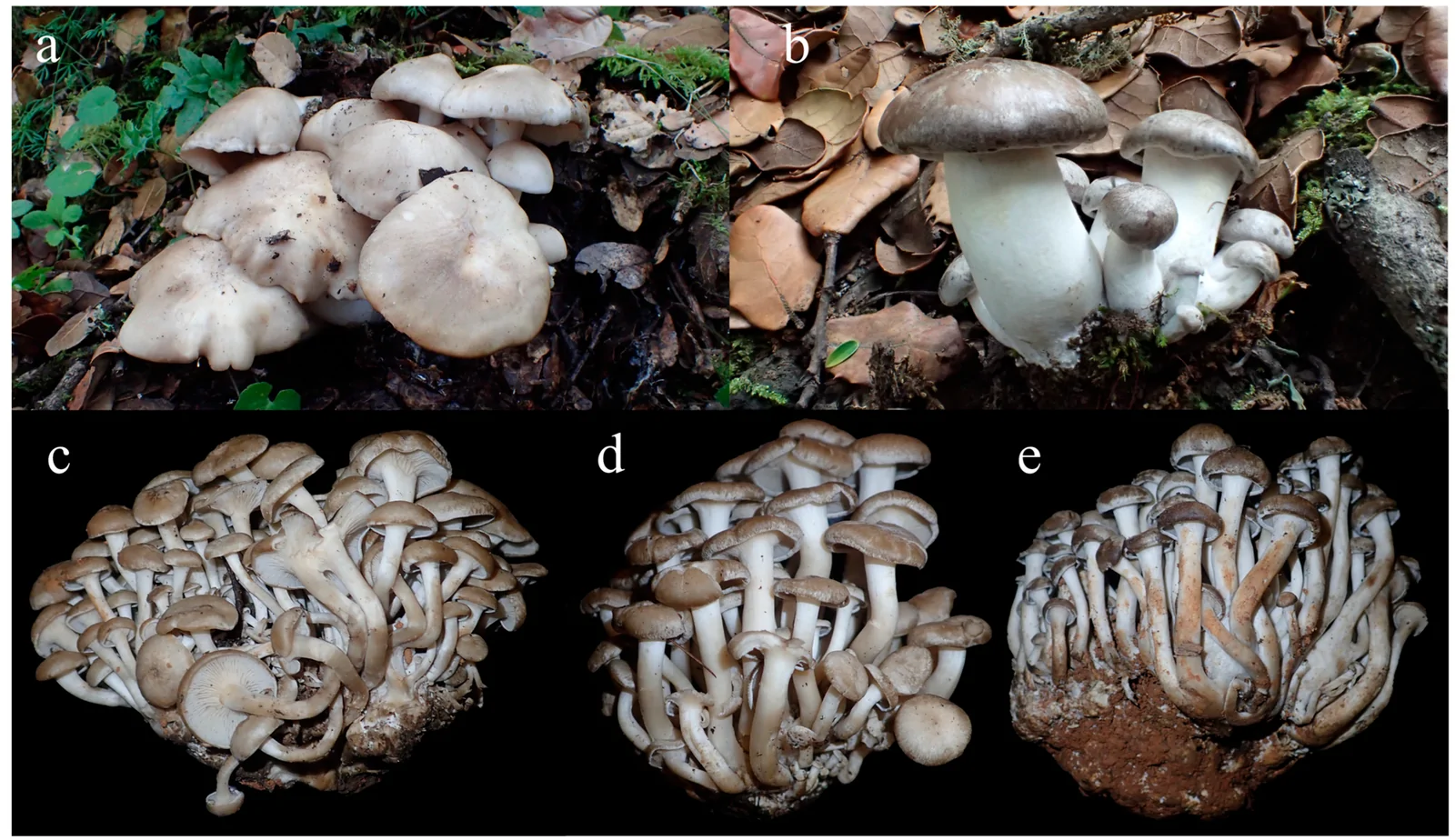
Field photographs of selected basidiomata assigned to the *Lyophyllum decastes* complex. (**a**,**b**) photographed in situ during field collection; (**c**–**e**) photographed indoors after field collection.

Occurrence records assigned to the *L. decastes* complex were compiled from the authors’ field surveys conducted since 2019, published reports, and academic monographs (Figure 1) [41]. Coordinates reported in non-decimal formats were converted to decimal degrees with the two-step outdoor assistant application [44]. We initially assembled 161 records and removed exact duplicates (Supplementary Table S1). The identification basis and availability of voucher or sequence data differed between sources. Raw molecular data are available only for the subset collected during the authors’ field surveys and will be supplied with the corresponding voucher metadata in the Supplementary Materials; sequence data were not available for all the literature-derived records. Consequently, we did not assign the pooled records to molecular operational taxonomic units and do not claim that all records represent a single lineage. In this study, “*L. decastes* complex” is an operational occurrence label encompassing records reported or morphologically assigned to the complex; the model estimates their pooled aggregate climatic envelope.

**Figure 2:**
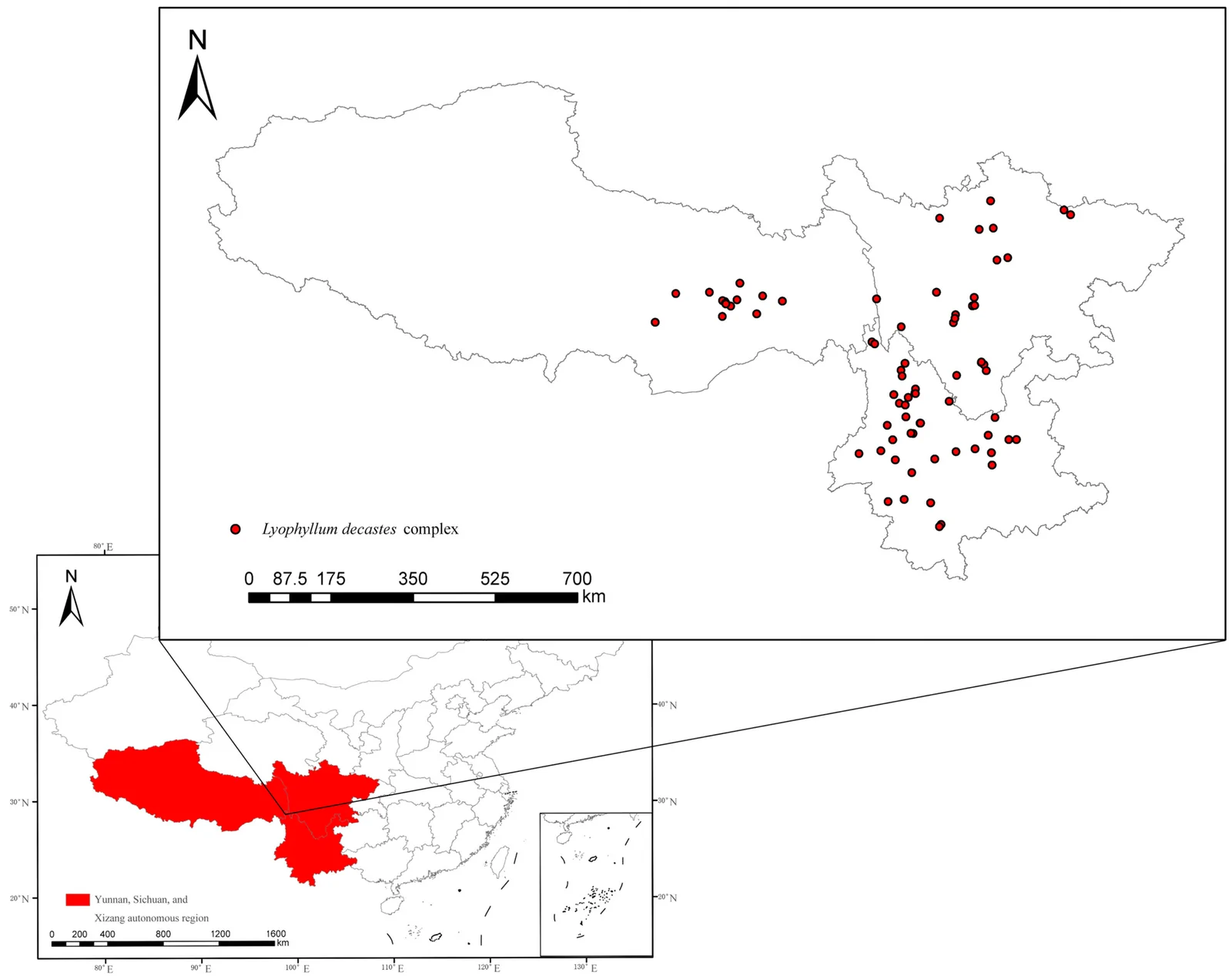
Geographic locations of *Lyophyllum decastes* complex distribution sites.

ENMTools software, released in 2010 by Warren et al. [45], was used to reduce spatial duplication in the occurrence dataset. Records falling within the same environmental grid cell were removed with the “Trim duplicate occurrences from .csv files” function in ENMTools v1.3. This procedure yielded 70 spatially thinned occurrence records for MaxEnt analysis (Figure 2, Supplementary Table S2). Spatial thinning reduces local clustering and potential overfitting but does not resolve the taxonomic heterogeneity of pooled records [46].

### 2.2. Acquisition and Preprocessing of Environmental Factors

The Intergovernmental Panel on Climate Change (IPCC) has highlighted in its Fifth Assessment Report that the global climate is currently exhibiting a pronounced warming trend. Under such circumstances, species distribution, population sizes, and genetic diversity are all subject to change. Environmental variables are pivotal in influencing the distribution of the *Lyophyllum decastes* complex. This chapter selects 19 bioclimatic variables (bio1-bio19) from the WorldClim database at a spatial resolution of 10 m [47]. Future climate data were determined based on the CCSM4 model for the decades of 2050 and 2070 [48]. The study adopts the four emission scenarios mentioned in the IPCC’s Fifth Assessment Report, which range from low to high as RCP2.6, RCP4.5, RCP6.0, and RCP8.5. Subsequently, the environmental factors are converted into ASCII format using the conversion tools within the ArcGIS software (version 10.8; Esri, Redlands, CA, USA).

This chapter integrates 70 sampling points, selected based on various environmental factors, into the local MaxEnt v3.4.4 software to predict the geographical distribution of the *L. decastes* complex in the regions of Sichuan, Yunnan, and Xizang. The software employs the built-in “Iterative Algorithms” parameter to calibrate the environmental factors, ultimately calculating the contribution rates of each environmental factor to the simulation results in the southwestern provinces of China [49]. To more distinctly characterize the performance of the *L. decastes* complex in terms of suitable climatic factors in this area, the output includes the six environmental factors with the highest contribution rates, which are annual mean temperature, the highest temperature of the warmest month, the lowest temperature of the coldest month, the average temperature of the coldest season, the precipitation of the wettest month, and the average temperature of the wettest season. Subsequent analyses focused on these six dominant climatic variables. Response curves were generated to examine their statistical associations with the model’s output, and climatic conditions corresponding to relative climatic-suitability values greater than 0.5 were provisionally identified.

### 2.3. Optimization and Statistical Evaluation of MaxEnt Models

We used MaxEnt v3.4.4 to characterize the aggregate climatic envelope of the pooled *L. decastes* complex occurrences across Sichuan, Yunnan, and Xizang. Before final model fitting, the regularization multiplier (RM) and feature-class (FC) combinations were tuned to balance fit and complexity. RM was varied from 0.5 to 4.0 in increments of 0.1, and seven FC combinations were evaluated with the ENMTools package in R v3.6.3. Candidate models were compared with AICc, where delta AICc = 0 identified the best-supported setting and delta AICc ≤ 2 indicated competing support. The selected setting produced an AUC of 0.953 and delta AICc = 0 after ten repeated runs. These metrics evaluate complexity and statistical discrimination for the pooled occurrence dataset; they do not establish taxonomic validity or lineage-specific predictive accuracy.

### 2.4. MaxEnt Model Selection and Evaluation

Utilizing the Spatial Analyst module within ArcGIS software, environmental factor values were extracted for each sampling point. When the correlation coefficient between two environmental variables exceeded 0.9, the environmental variable with lesser biological significance within the highly correlated group was eliminated, culminating in the construction of a model with the selected environmental variables.

After processing the distribution and environmental variable data, model building was carried out using MaxEnt v3.4.4 by first randomly selecting 25% of the distribution points as a test dataset, with the remaining 75% serving as the training dataset; setting the maximum number of iterations of the model to 500; establishing the number of repetitions to 10; opting for cross-validation as the method of repetition; employing the software’s Jackknife test to calculate the contribution of environmental variables; and then exporting the predicted results in ASCII format.

### 2.5. Ecological Niche Analysis

The MaxEnt raster output was interpreted as relative climatic suitability on a scale from 0 to 1 and not as an individual survival probability or confirmed occupancy. For map summaries, climatic suitability was stratified into five classes: high (P ≥ 0.75), medium-high (0.55 ≤ P < 0.75), medium (0.30 ≤ P < 0.55), low (0.13 ≤ P < 0.30), and unsuitable (P < 0.13). Higher values indicate stronger similarity to the climatic conditions associated with the pooled occurrence records; they do not demonstrate host presence, lineage identity, or realized habitat suitability.

For change analysis, cells with climatic suitability ≥ 0.50 were provisionally classified as climatically suitable while lower values were climatically unsuitable. Binary maps were compared among periods to identify newly suitable, retained, and lost climate space. These categories describe the changes in modeled climate space only and should not be interpreted as confirmed colonization, persistence, or local extinction.

### 2.6. Centroid Migration and Areal Statistics of Ecological Niches

We have compiled the grid conditions of the *L. decastes* complex across various suitable habitat zones in Sichuan, Yunnan, and Xizang under four distinct emission scenarios. Utilizing Excel 2021, we calculated the proportion of pixels representing different levels of habitat suitability. We first extracted the terrestrial land area of China that corresponds to the regions of Sichuan, Yunnan, and Xizang and then converted the raster pixels of the potential suitable distribution area of the *L. decastes* complex into the actual terrestrial land area they occupy.

Species can exhibit varying trends in the spatial shift in their suitable distribution areas under climate change conditions. The study of centroid displacement offers a visually intuitive representation of these changes. Given the irregular shape of the suitable distribution range of the *L. decastes* complex, we define the geographical center of the suitable habitat as the central point of the complex’s suitability changes in Sichuan, Yunnan, and Xizang over different periods. The migration of the suitable distribution range across different periods and scenarios is represented by the coordinates of the centroid.

## 3. Results

**Figure 3:**
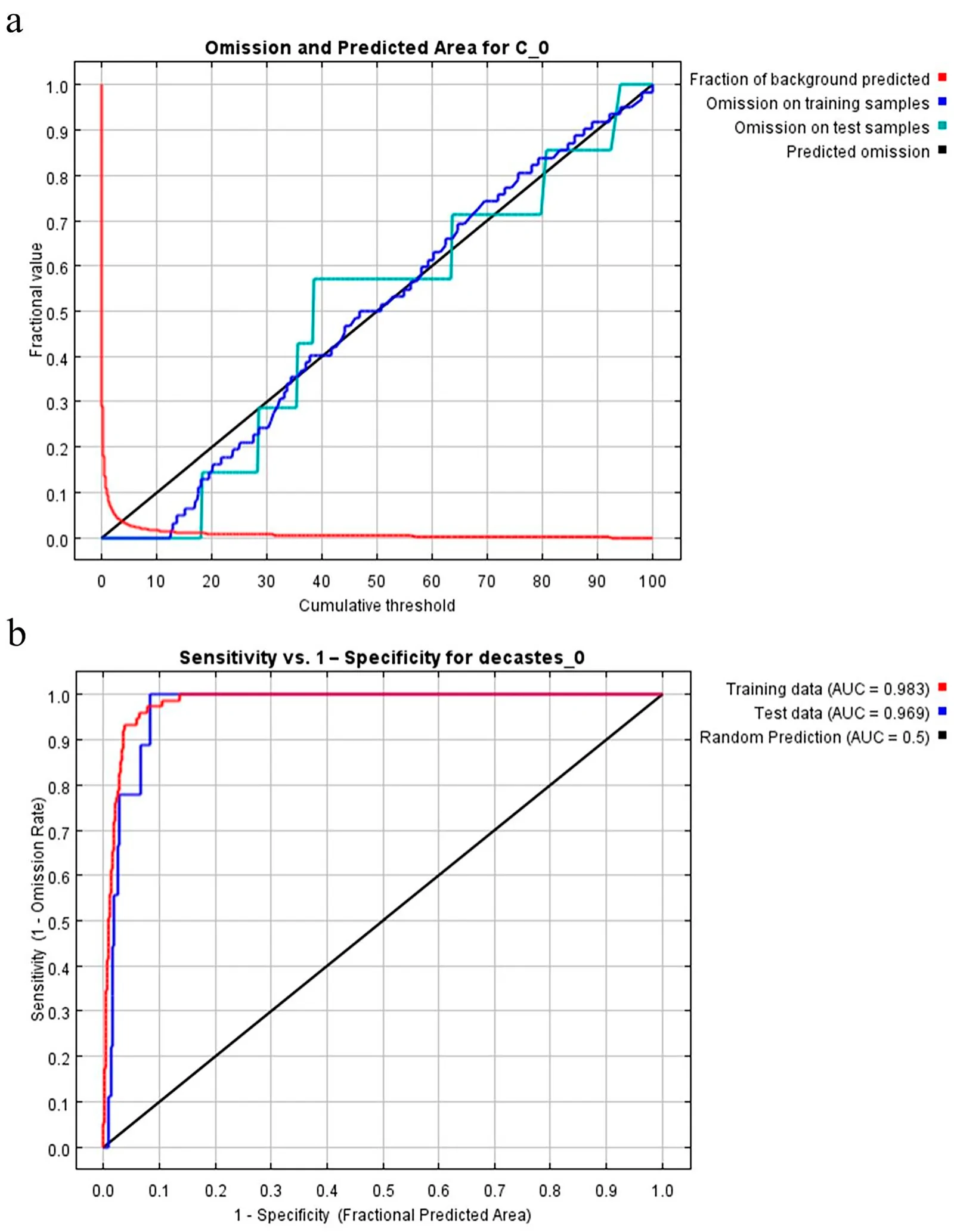
Maximum entropy model repeated running ROC curve. (**a**) The average value of the ROC curve of the MaxEnt model repeated 10 times; and (**b**) the ROC curve of the ninth run of the MaxEnt model.

We utilized the MaxEnt model to simulate the suitable distribution range of the *L. decastes* complex in Sichuan, Yunnan, and the Xizang Autonomous Region of China. Using the default parameters, the delta AICc value was 274.97. When the model parameters were adjusted to set FC to QP and RM to 3.6, the delta AICc value was reduced to zero. Comparative analysis revealed that, by refining the model parameters with the ENMtools package in R, the model’s overfitting and complexity were both lower than those with the default parameters of this study’s model. Consequently, we adopted FC set to QP and RM set to 3.6 as our modeling parameters. To evaluate discrimination under the optimized settings, we resimulated the suitable distribution range of the *L. decastes* complex using the optimized parameter settings and obtained the training and testing AUC values from the dataset through cross-validation (Figure 3). The results demonstrated that after ten simulations, both the training and testing AUC values exceeded 0.95, indicating strong discrimination for the pooled occurrence dataset. Upon comparing these ten sets of simulations, the ninth set yielded test and training AUC values of 0.969 and 0.983, respectively. Therefore, we selected this particular set for further adaptiveness analysis.

### 3.1. Geographical Distribution and Environmental Correlation of the L. decastes Complex

**Figure 4:**
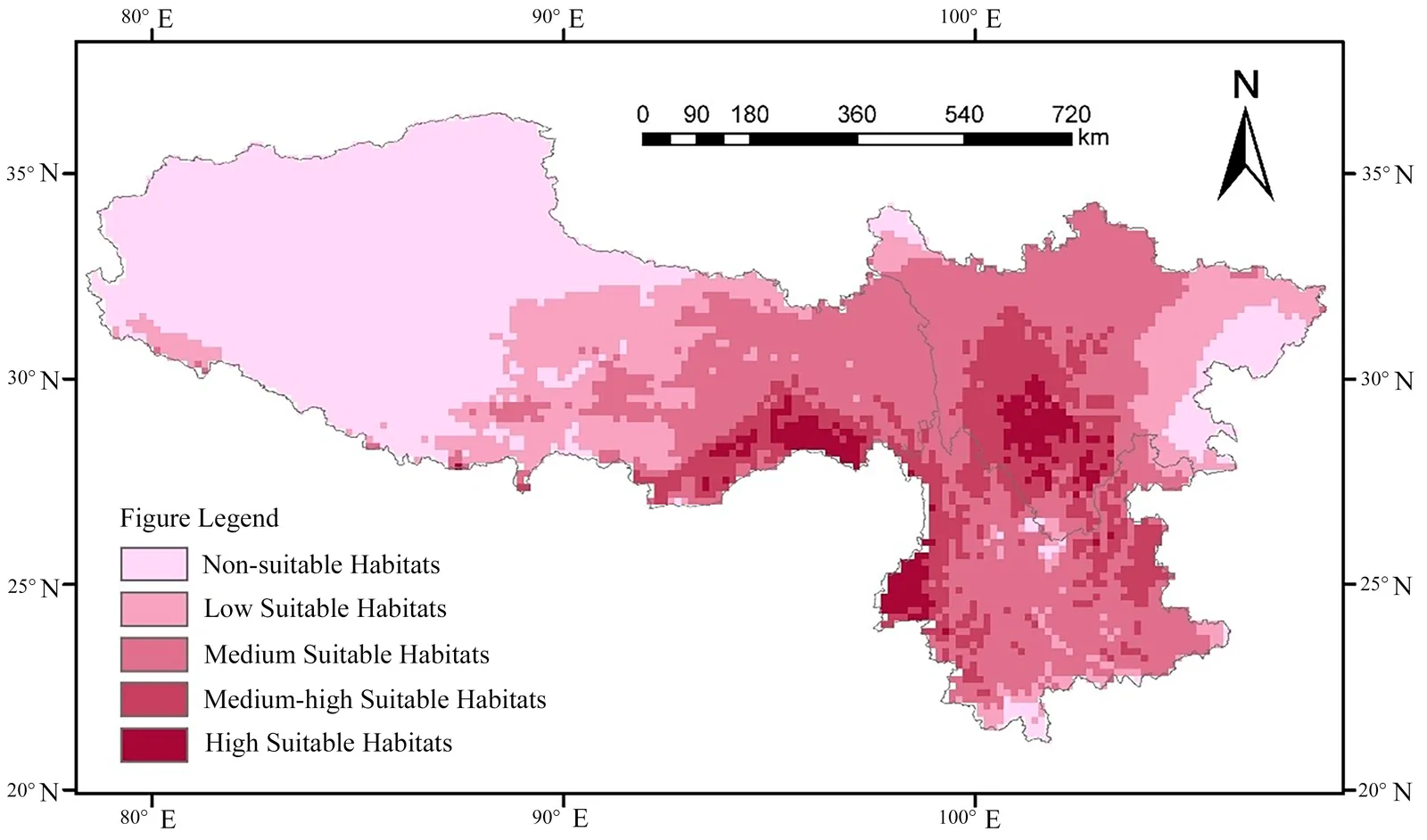
Potential distribution of *Lyophyllum decastes* complex in Yunnan, Sichuan, and the Xizang Autonomous Region under current climatic conditions.

Utilizing an optimized MaxEnt model based on 70 georeferenced data points of the *L. decastes* complex and six predominant climatic variables, we simulated the potential distribution areas under current climatic conditions for Sichuan, Yunnan, and the Xizang Autonomous Region (Figure 4). Concurrently, we employed the internationally recognized Jenks natural breaks method to categorize the suitable habitats from low to high into various grades within these regions. In this study, “highly suitable habitat” refers to areas with high climatic similarity to known occurrence localities of the *L. decastes* complex, as estimated by the MaxEnt model and classified using the Jenks natural breaks method, rather than confirmed realized habitat, because vegetation type, soil properties, substrate availability, slope, microhabitat humidity, and other local ecological factors were not directly included as predictors. The stratification outcomes were subsequently imported into the ArcGIS software to delineate the suitable distribution areas of the *L. decastes* complex under current climatic scenarios. Our analysis revealed that the suitable distribution range of the *L. decastes* complex in these regions is irregularly shaped and predominantly concentrated in most areas of Sichuan except the northeastern part, the entire area of Yunnan (excluding the southern part), and the eastern part of the Xizang Autonomous Region.

Highly suitable habitats are predominantly found in the northwestern region of Yunnan Province, the central and southern regions of Sichuan Province, and the southeastern part of the Xizang Autonomous Region, with specific distribution sites detailed in Table 1. Moderately high and moderate suitable habitats radiate outward from the highly suitable areas. Based on the grid data of the suitable distribution range for the *L. decastes* complex, we calculated the area of suitable distribution across the three provinces. The results indicate that under current climatic patterns, the area of highly suitable distribution zones accounts for 7.1 × 10^4^ km^2^, representing approximately 3.41% of the total terrestrial ecological area of the three provinces. This suggests that the highly suitable range for the *L. decastes* complex occupies a relatively small proportion and is dispersed across these provinces. The areas of moderately high and moderate suitable zones are 24.5 × 10^4^ km^2^ and 66.0 × 10^4^ km^2^, respectively, constituting 11.76% and 31.69% of the total terrestrial ecological area of the three provinces. The area with low-suitable habitats is 33.1 × 10^4^ km^2^, which accounts for 15.89% of the total terrestrial ecological area. The total suitable range under current climatic conditions is approximately 129.6 × 10^4^ km^2^, representing about 62.22% of the total land area of the three provinces.

### 3.2. Key Environmental Drivers of L. decastes Complex Distribution

**Figure 5:**
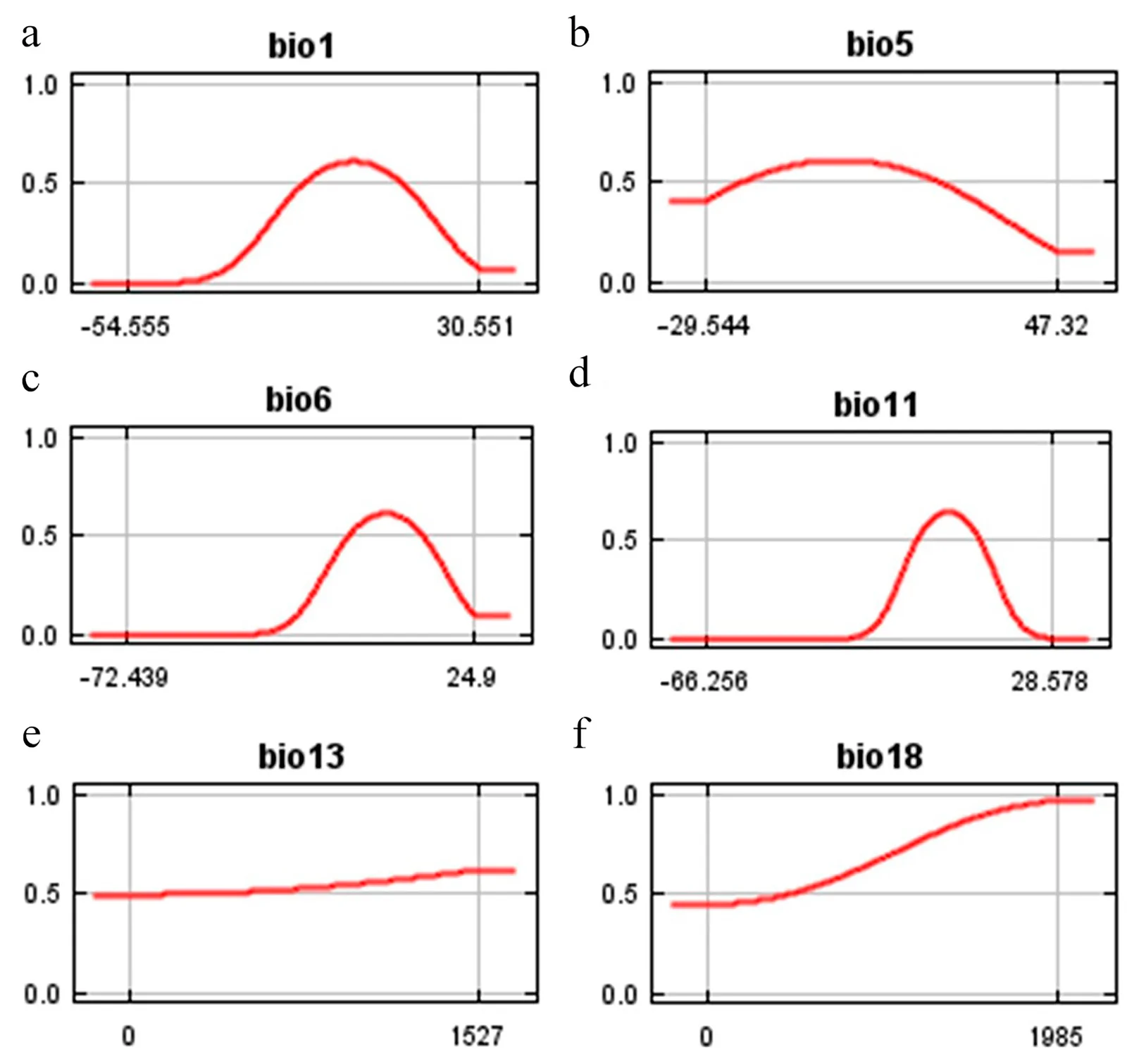
Relationships between the potential geographical distribution of the *Lyophyllum decastes* complex and the dominant climate factors. (**a**) Annual mean temperature; (**b**) max temperature of warmest month; (**c**) min temperature of coldest month; (**d**) mean temperature of coldest quarter; (**e**) precipitation of wettest month; and (**f**) precipitation of warmest quarter.

We used response curves and permutation-based summaries to describe statistical associations between the pooled occurrence dataset and the six retained climatic variables. The mean temperature of the coldest quarter, maximum temperature of the warmest month, minimum temperature of the coldest month, mean temperature of the wettest quarter, precipitation of the wettest month, and annual mean temperature contributed 50.8%, 26.1%, 11.9%, 9.1%, 1.8%, and 0.2%, respectively (Figure 5). These percentages describe the fitted aggregate model and should not be interpreted as physiological tolerances shared by every lineage. In particular, the 50.8% contribution may partly reflect the taxonomic composition, geographic clustering, and high-elevation sampling structure of the pooled records.

### 3.3. Impact of Future Climate Change on the Ecological Niche Distribution of the L. decastes Complex

**Figure 6:**
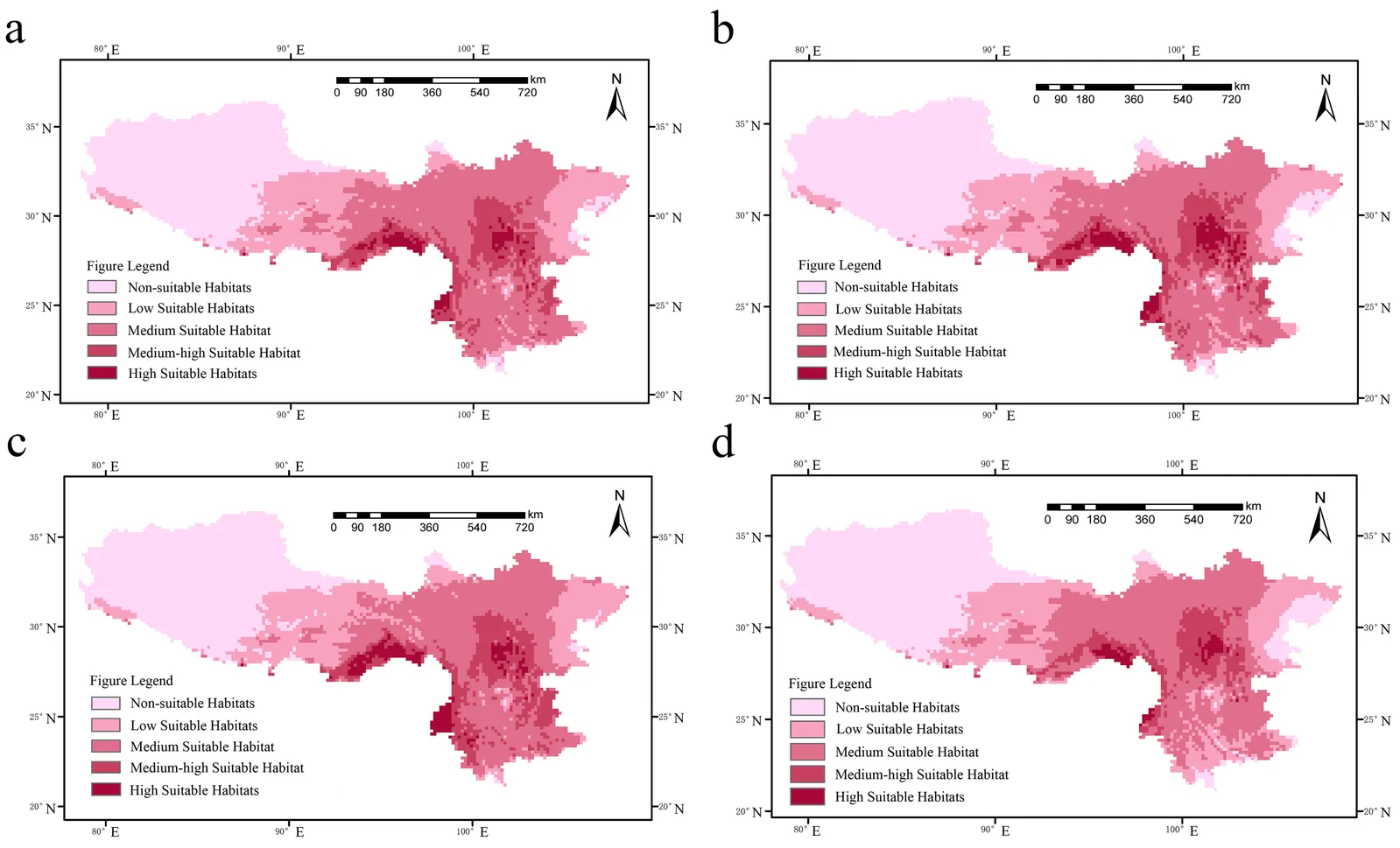
Potential distribution of the *Lyophyllum decastes* complex in the 2050s. (**a**) Potential distribution area of the species complex in the 2050s under RCP2.6; (**b**) potential distribution area of the species complex in the 2050s under RCP4.5; (**c**) potential distribution area of the species complex in the 2050s under RCP6.0; and (**d**) potential distribution area of the species complex in the 2050s under RCP8.5.

**Figure 7:**
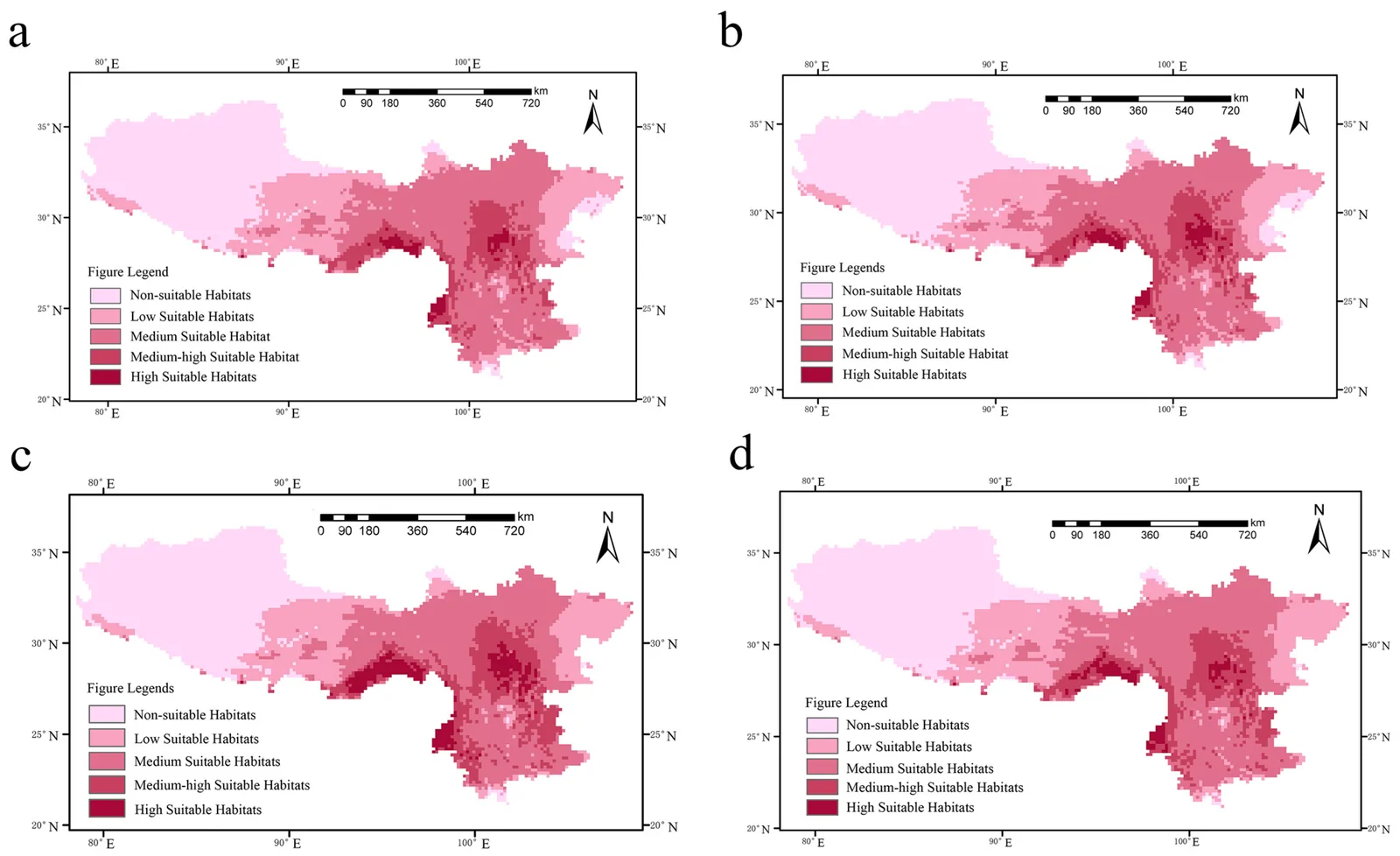
Potential distribution of the *Lyophyllum decastes* complex in the 2070s. (**a**) Potential distribution area of the *Lyophyllum decastes* complex in the 2070s under RCP2.6; (**b**) potential distribution area in the 2070s under RCP4.5; (**c**) potential distribution area in the 2070s under RCP6.0; and (**d**) potential distribution area in the 2070s under RCP8.5.

By integrating the distribution points of the *L. decastes* complex with future climatic scenarios and geographically distributed environmental factors over the next century, we project its suitable habitat range under RCP2.6, RCP4.5, RCP6.0, and RCP8.5 for the 2050s and 2070s. As indicated in Figure 6 and Figure 7, the distribution ranges of non-, low-, medium-, medium-high-, and high-suitable habitats for this complex are expected to shift under various future climate scenarios compared to current conditions, with the most significant changes occurring in a gradual distribution in the central-eastern and southwestern regions. In the 2050s and 2070s, under different climate scenarios, the most pronounced changes in the suitable range of the *L. decastes* complex, relative to the current distribution, are observed in the Ganzi Tibetan Autonomous Prefecture of Sichuan and the western part of Yunnan. Under the RCP2.6 scenario, these areas exhibit a decline in habitability, with a significant reduction in high-suitable habitats and a transition from non-suitable to low- and medium-suitable habitats. In Xizang, high-suitable habitats are projected to decrease from the 2050s to the 2070s, with low-suitable habitats spreading eastward. Under the RCP4.5 scenario, high-suitable habitats are expected to slightly increase and become more fragmented by the 2050s and 2070s, with no significant change in medium-suitable habitats and a trending increase in low-suitable habitats. Compared to RCP2.6, the overall suitable distribution area under RCP4.5 shows a gradual decrease, with high-suitable habitats being smaller than under current conditions, while medium- and low-suitable habitats expand to varying degrees. In the RCP6.0 scenario, high-suitable habitats are projected to significantly increase and become more concentrated by the 2050s and 2070s, with a slight increase in low-suitable habitats and little change in the central suitable habitats. Under RCP8.5, the suitable distribution area is expected to markedly decrease compared to current conditions, with an increase in low-suitable and non-suitable habitats, while the central suitable habitats remain largely unchanged (Table 2).

### 3.4. Impact of Future Climate Patterns on Ecological Niche Shifts in the L. decastes Complex

**Figure 8:**
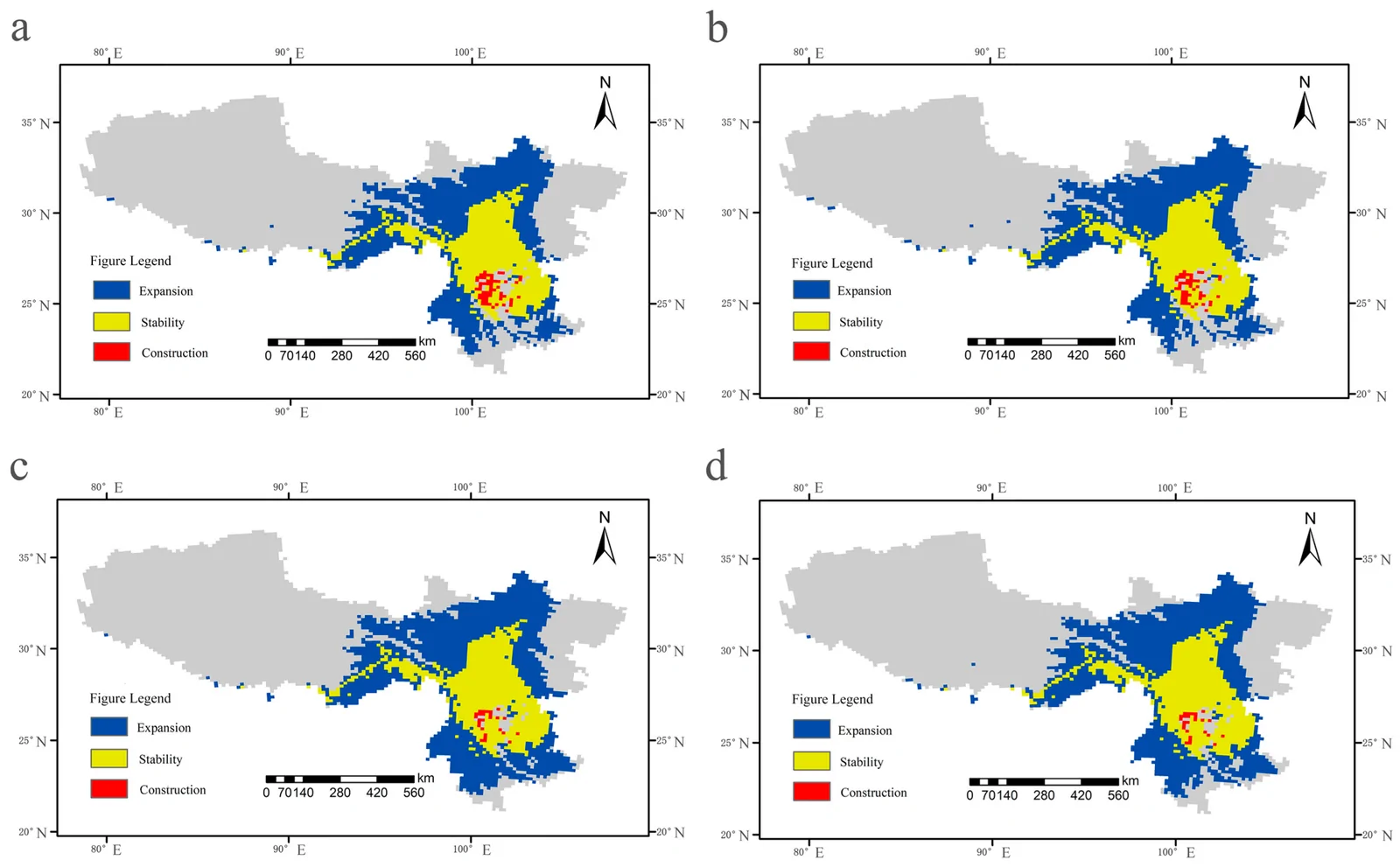
Shift in suitable habitats for the *Lyophyllum decastes* complex under different climate scenarios in the 2050s. (**a**) Changes in the potential distribution in the 2050s for RCP2.6; (**b**) changes in the potential distribution of under the 2050s for RCP4.5; (**c**) changes in the potential distribution of under the 2050s for RCP6.0; and (**d**) changes in the potential distribution of in the 2050s for RCP8.5.

**Figure 9:**
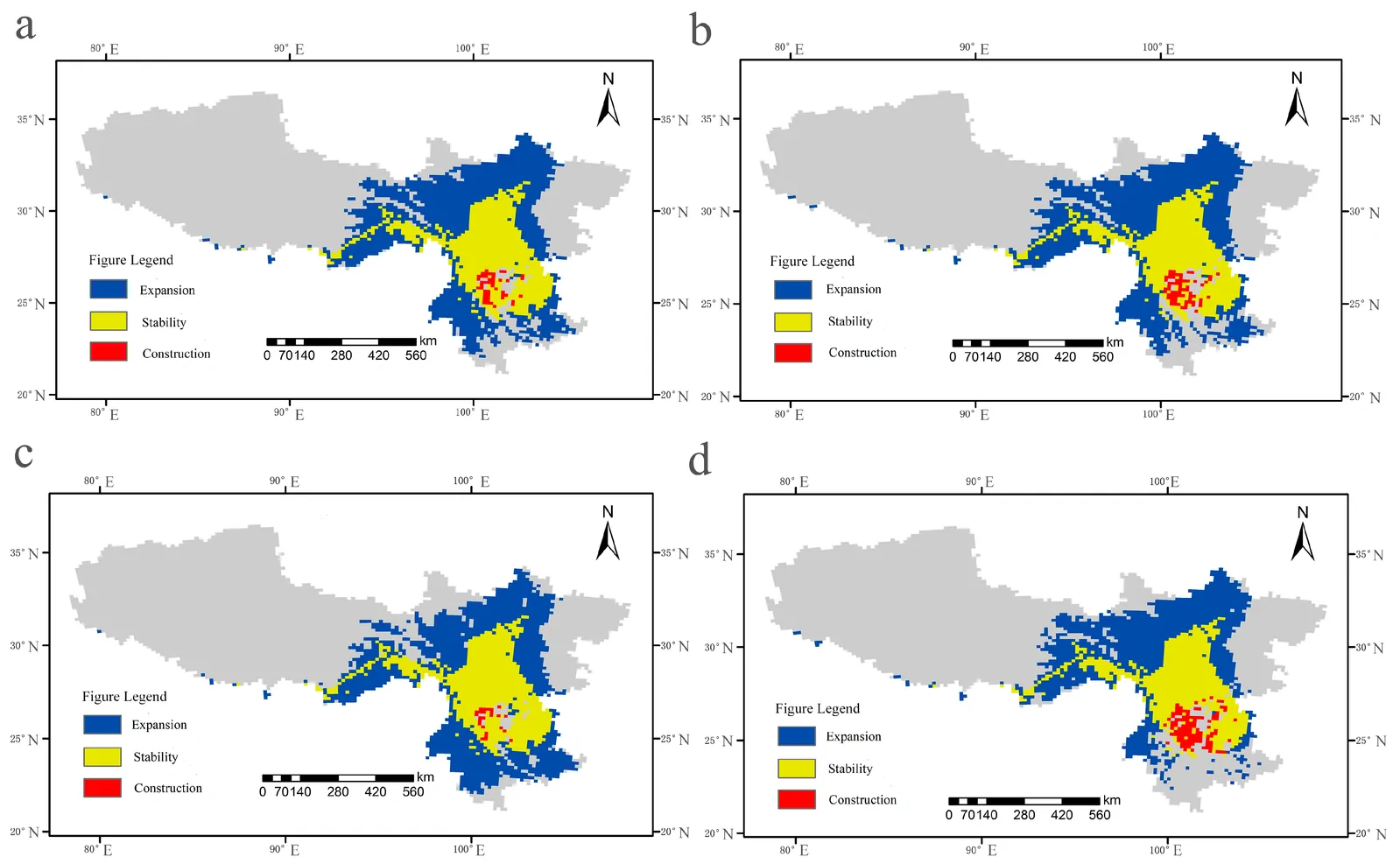
Characteristics of suitable habitat transfer of the *L. decastes* complex under different climate scenarios in the 2070s. (**a**) Changes in the potential distribution of the species complex in the 2070s for RCP2.6; (**b**) changes in the potential distribution of in the 2070s for RCP4.5; (**c**) changes in the potential distribution of in the 2070s for RCP6.0; and (**d**) changes in the potential distribution of in the 2070s for RCP8.5.

We have calculated the suitable and unsuitable habitats for the *L. decastes* complex in Sichuan, Yunnan, and Xizang under current and future climate scenarios, and assessed the spatial shifts in the *L. decastes* complex within these regions under various future climate patterns. Additionally, we have evaluated whether the area will experience habitat loss, gain, or remain unchanged (Table 3, Supplementary Table S4) based on the distribution of pixel points and the terrestrial land area of the Yunnan–Sichuan–Xizang region. This assessment aims to determine the potential impacts of climate change on the *L. decastes* complex under increasing carbon emissions by examining changes in suitable habitats. The central suitable distribution areas for the *L. decastes* complex under different climate scenarios in the 2050s exhibit a loss of suitable habitats (Figure 8). Within the overall framework, new suitable habitats appear on the eastern and western sides in the south, with an irregular pattern of addition being the predominant trend. The spatial shifts in the complex under various climate scenarios in the 2070s (Figure 9) are similar to those in the 2050s, indicating new habitat additions in the southern and northwestern regions, along with an irregular pattern of newly suitable areas.

### 3.5. Centroid Shifts and Ecological Niche Dynamics of the L. decastes Complex Under Future Climate Scenarios

Given the irregular distribution of the *L. decastes* complex, it is imperative to employ the centroid to delineate the central point of potential suitable growth environments for this composite group. Consequently, this section calculates the centroid distribution of the *L. decastes* complex in Sichuan, Yunnan, and Xizang under current and future climate scenarios at different time periods (Table 4), aiding in the interpretation of potential shifts in suitable growth environments and migration patterns under future climatic conditions.

The centroid of the potential suitable habitat for the *L. decastes* complex under current climatic conditions is located at (100°39′22″ N 27°37′12″ E) in Yongning Town, Ninglang Yi Autonomous County, Lijiang City, Yunnan Province. Under the RCP2.6 emission scenario, the centroid remains unchanged in Yongning Town for both the 2050s and 2070s. Under the RCP4.5 scenario, the centroid shifts from Yongning Town (100°39′22″ N 27°37′12″ E) to (99°47′24″ N 28°35′24″ E) in Dongwang Township, Shangri-La City, Diqing Tibetan Autonomous Prefecture, Yunnan, marking a migration of 127.78 km in the 2050s. By the 2070s, the centroid further migrates to (99°49′48″ N 28°33′18″ E), also in Dongwang Township, with a distance of 120.97 km from the current climatic pattern. Under the RCP6.0 scenario, the centroid moves from Yongning Town (100°39′22″ N 27°37′12″ E) to (100°1′12″ N 28°16′12″ E) in Ceta Lai, Shangri-La City, Diqing Tibetan Autonomous Prefecture, Yunnan—a distance of 85.43 km—in the 2050s. By the 2070s, it shifts to (100°15′36″ N 27°58′11″ E) in Du Ran, Shangri-La City, with a migration of 46.80 km from the current climatic pattern. Under the RCP8.5 scenario, the centroid migrates from the current climatic pattern to (99°57′35″ N 28°18′36″ E) in Songdu Ga Zha, Shangri-La City, Diqing Tibetan Autonomous Prefecture, Yunnan—a distance of 128.18 km—in the 2050s. In the 2070s, under the same scenario, the centroid shifts to (99°31′48″ N 29°4′48″ E) in Yongde Village, Zhengdou Township, Xiangcheng County, Ganzi Prefecture, Sichuan. The centroid position of *L. decastes* complex gradually migrates northwestward with increasing carbon emissions within the same period across different emission scenarios.

## 4. Discussion

To elucidate the potential factors influencing the distribution of the *L. decastes* complex, it is essential to identify suitable habitat characteristics by integrating diverse environmental data. This endeavor must also confront the challenges posed by urban expansion, which affects species distribution and the reduction in suitable habitats [50,51,52,53]. Predicting the dispersal of such populations is crucial for devising conservation and reintroduction strategies. Species distribution models (SDMs) offer a methodology to identify potential geographic distribution patterns of species. Among the developed SDMs are Generalized Linear Models (GLMs) [54]; Artificial Neural Networks; Random Forests (RFs) [55]; and the MaxEnt model, which performs well with small sample sizes or limited data, and is considered a relatively robust model.

This study collates data on the distribution of the *L. decastes* complex in Sichuan, Yunnan, and Xizang from multiple perspectives and dimensions. We comprehensively selected two parameters, delta AICc and AUC, based on the actual distribution of the *L. decastes* complex. The results indicate that when the Response Mode (RM) value is 0.4, the AUC value is at its peak; however, the delta AICc value is also high, suggesting a high complexity of the parameter model. As the RM value increases, the delta AICc decreases, reducing the model’s complexity, but the AUC also tends to decline. When the delta AICc is less than two, there are two optimal parameter settings: one with RM at 0.1 and the Feature Mode (FM) set to LQ, and the other with RM at 3.6 and FM set to QP. Therefore, when considering all factors, this study selects the parameter setting where delta AICc is zero, RM is 3.6, and FM is QP.

Correlation filtering and parameter optimization reduced collinearity and model complexity. The optimized MaxEnt model had an average AUC above 0.95 across ten iterations, supporting strong discrimination for the pooled occurrences. This result does not demonstrate biological precision, taxonomic homogeneity, or accurate lineage-specific distributions; it supports only the internal statistical performance of the aggregate model.

The pooled model identifies broad areas whose climates resemble those associated with known records of the *L. decastes* complex. Agreement between parts of the projected envelope and known occurrences supports its use as a survey-prioritization tool, but it does not constitute taxon-level validation. Predicted areas without records may reflect incomplete sampling, limited dispersal, absence of required biotic partners, taxonomic pooling, or extrapolation beyond the sampled environmental domain. Likewise, the response curves are associations within the fitted model rather than direct estimates of survival. The apparent importance of cold-quarter temperature may be biologically plausible for high-elevation records, but the present data cannot establish whether it applies to all cryptic lineages in the complex.

Our study investigates the suitable distribution range of the *L. decastes* complex under current climatic conditions in Sichuan, Yunnan, and Xizang, China. The findings reveal that the suitable habitats under the current climate are predominantly located in the majority of Yunnan, central and central-western regions of Sichuan, and the southeastern part of the Xizang Autonomous Region. A small number of suitable habitats are also found in the western part of Xizang, corroborating previous surveys [42] and validating the precision of our method in predicting the distribution of the *L. decastes* complex in this area. The simulated suitable distribution area includes existing locations, aligning with previous biodiversity research [29,56]. The predictions indicate the presence of medium- and low-suitable habitats for the complex in the northern and northeastern parts of Xizang, yet no distribution records have been found in these areas. This may be attributed to the underexplored nature of these regions, necessitating further research and supplementary sampling [57]. Another potential factor is the limited migration capacity of the *L. decastes* complex, hindering the species’ ability to move to suitable environments, thus creating a discontinuity in the central region [58]. The contribution rate of environmental factors shows that the average temperature of the coldest season, the highest temperature of the hottest month, and the lowest temperature of the coldest month are the dominant climatic factors affecting geographical distribution, with a cumulative contribution rate of 87.1%. Notably, the average temperature of the coldest season has the highest contribution at 50.8%, suggesting its significant role as a potential environmental factor influencing the distribution of the *L. decastes* complex in Yunnan, Sichuan, and Xizang. As depicted in Figure 5, the survival probability of the *L. decastes* complex is zero when the average temperature of the coldest season is below −38 °C, and it significantly increases with rising temperatures. The highest survival probability occurs when the average temperature is around 0 °C, after which it gradually declines, and almost reaches zero when the average temperature exceeds 28 °C. This indicates the complex’s sensitivity to temperature changes and a significant increase in survival probability with increasing average temperatures of the coldest season. When the highest temperature of the hottest month is below 26 °C, the survival probability remains suitable, and it gradually decreases thereafter. When the precipitation of the wettest month exceeds 200 mm, the survival probability is at a suitable level, indicating the complex’s strict requirements for environmental humidity. A comprehensive analysis of the environmental factor response curves reveals that the suitable range for the coldest season’s average temperature is between −12 °C and 12 °C, for the hottest month’s highest temperature between −25 °C and 26 °C, and for the coldest month’s lowest temperature between −10 °C and 10 °C. These modeled associations are broadly consistent with the occurrence of many records in relatively cool and moist environments [59]. However, the present data cannot establish that these climatic responses apply uniformly to every lineage within the *L. decastes* complex.

Over the forthcoming century, the incremental warming of the climate and the relentless rise in atmospheric temperatures are poised to exert a variety of impacts on the adaptive distribution of species, with the majority experiencing a gradual decline in environmental adaptability as temperatures soar. Steidinger et al. [33] projected the future of ectomycorrhizal fungi in North American coniferous forests, revealing a potential decline in species richness of up to 26%, a rate that can be moderated by reducing greenhouse gas emissions. Wei et al. [60] explored the implications of future climate change on *Ophiocordyceps sinensis* and found no significant changes under the RCP2.6 scenario; however, under RCP4.5, RCP6.0, and RCP8.5 scenarios, there was a spatial shift and a marked reduction in suitable growth areas in the Tianshan, Kunlun, and Southwestern Tibetan Plateau regions. Guo et al. [39] investigated the geographical distribution of *Polyporus umbellatus* across various climate scenarios in China, demonstrating that annual precipitation and mean annual temperature largely determine its suitable distribution, suggesting that *Polyporus umbellatus* growth may require a humid and shady environment, with its distribution often associated with the complex diversity of forest types. Cao et al. [40] studied the suitable habitats of *Morchella* in China, identifying Yunnan, Sichuan, Gansu, Shaanxi, and the Xinjiang Uyghur Autonomous Region as primary distribution areas, also noting a future trend of expansion towards the northeast and northwest regions. Santos et al. [38] analyzed the suitability factors for *Auricularia brasiliana* Y.C. Dai & F. Wu in Wu, Yuan, Vlasák, Rivoire & Dai and *Megasporoporia neosetulosa* C.R.S. de Lira & Gibertoni, as well as in Lira, Alvarenga, Soares, Ryvarden & Gibertoni, indicating that temperature and precipitation strongly influence the future persistence of these species, with a gradual reduction in tropical regions also expected due to climatic impacts.

In recent years, by examining the meteorological data of our country, similar trends have been observed both domestically and globally, with a gradual increase in surface temperatures from south to north, an exacerbation of extreme heat, and a reduction in extreme cold events [61]. The natural resources of the wild *L. decastes* complex are limited, not only due to their stringent environmental requirements and restricted geographical distribution but also because of overexploitation by local residents in recent years, which poses a severe threat to their wild populations and sustainable occurrence [62]. Consequently, this research reveals significant changes in the geographical distribution of the complex in Sichuan, Yunnan, and Xizang under various future climate scenarios compared to the present, yet with minimal changes in total area. Under warming conditions, the area of low suitability for the complex in these regions tends to expand, while the area of moderate suitability shows little change, and the areas of medium-high and high suitability are on the decline.

The evaluation of the model confirms the robustness of the current predictive results [63]. However, several crucial factors affecting the potential distribution of species have not yet been considered, including external environmental factors, such as soil pH, slope orientation data, and vegetation type data across different regions, as well as other factors like changes in land use, ecological niche competition, and human mediation. Additionally, the species’ own dispersal capabilities can influence their distribution range. Therefore, these factors will become the subject of future research and will be included as model parameters to enhance the accuracy and reliability of determining species’ adaptation to environments and the construction of habitat systems.

The principal limitation of this analysis is that the 70 occurrences were not uniformly defined with ITS, LSU, or multilocus data. Our field-collected subset has associated raw molecular data, whereas most records compiled from external publications lack comparable sequences or vouchers (Supplementary Table S3). Selective molecular confirmation of only one subset cannot resolve the identity of all pooled records. The modeling unit is therefore an operational complex and not a molecularly delimited species or OTU.

Pooling unresolved lineages can broaden or distort an estimated niche if lineages differ in environmental tolerance, trophic strategy, or sampling frequency. Consequently, the high AUC values and variable contributions may partly reflect mixture composition and sampling bias. The maps and numerical thresholds must not be extrapolated as lineage-specific physiological limits, population trends, extinction risks, or definitive conservation boundaries. Molecularly resolved occurrence records are required before taxon-specific models can be constructed.

Members of the broader complex may also differ in nutritional strategy and host association. In particular, *Lyophyllum shimeji*, a member of the *L. decastes* complex, has been experimentally confirmed as an ectomycorrhizal associate of conifer seedlings, including *Pinus pinaster* and *Picea abies* [43]. Such evidence shows that host availability and host migration can mediate realized occurrence, even where the climate appears suitable. Because the present model includes only bioclimatic predictors, it cannot separate direct climatic effects on fungi from indirect effects operating through host plants, vegetation, soils, land use, competitors, dispersal, or collection pressure. In particular, the vegetation type may determine host availability, litter composition, canopy cover, and local moisture conditions; soil properties may affect organic matter content, water retention, and fungal growth; slope and terrain may influence drainage, solar radiation, and microclimates; and substrate availability may directly affect saprotrophic members of the complex. These variables are therefore ecologically relevant for defining suitable habitats, even though they were not included as predictors in the present broad-scale climatic model. We therefore no longer describe temperature as the sole distribution driver. Future analyses should combine molecularly delimited lineages with host or vegetation layers, edaphic variables, substrate availability, slope or terrain variables, land-use change, bias-aware sampling, and dispersal constraints. Wild fruiting bodies assigned to the *L. decastes* complex are locally harvested, and collection pressures may interact with habitat change [62]. However, the present climate-only model does not estimate abundance, demographic decline, or extinction risk. Its conservation relevance is limited to identifying candidate areas for taxonomically stratified surveys, long-term monitoring, and evaluation of harvesting intensity.

## 5. Conclusions

The AICc-optimized MaxEnt model showed strong discrimination for the pooled occurrence records assigned to the *Lyophyllum decastes* complex, with mean AUC values above 0.95. Among the retained bioclimatic variables, mean temperature of the coldest quarter had the strongest statistical association with the fitted model, contributing 50.8%. The modeled climatic envelope was concentrated mainly in Yunnan, Central and Central-Western Sichuan, and Southeastern Xizang under current conditions. Projected changes varied among climate scenarios and periods, with increased fragmentation and reductions in medium-high or high climatic-suitability classes under several future scenarios. These results provide a regional climate-based baseline for identifying areas that may warrant further investigation. However, the model represents the aggregate climatic envelope of pooled records attributed to the *L. decastes* complex rather than the distribution or ecological niche of a single molecularly delimited species. The results should not be extrapolated as lineage-specific physiological tolerances, confirmed occupancy, population trends, colonization, local extinction, or definitive conservation boundaries. The principal limitations include the lack of uniform molecular verification across occurrence records; possible taxonomic and sampling heterogeneity; and the omission of host availability, vegetation, soil, substrate, land-use change, dispersal, competition, collection pressure, and other local ecological processes. Accordingly, the primary contribution of this study is to generate spatial hypotheses and identify candidate areas for taxonomically stratified field surveys, molecular sampling, host and substrate verification, and long-term habitat monitoring. Future lineage-specific assessments will require molecularly delimited occurrence records combined with relevant abiotic and biotic predictors.

## Figures and Tables

**Table 1:** The highly suitable distribution of the *L. decastes* complex in Sichuan, Yunnan, and the Xizang Autonomous Region of China.

Provinces	Main Distribution Areas
Yunnan Province	Lushui City, Fugong County, Tengchong City, Lianghe County, Yingjiang County, Longling County, and Longchuan County
Sichuan Province	Jiulong County, Mianning County, Yuexi County, Yanyuan County, Bushi County, and Zhaojue County
Xizang Autonomous Region	Nyingchi City, Milin County, Bomi County, and Medog County

**Table 2:** Suitable area of *L. decastes* complex under different climate scenarios (×10^4^ km^2^).

Different Climate	Period	High-Suitable Habitats	Medium-High-Suitable Habitats	Medium-Suitable Habitats	Low-Suitable Habitats
Current	1985s	7.05	23.47	65.99	33.07
RCP2.6	2050s	3.65	19.49	69.98	42.81
	2070s	4.71	21.37	68.39	42.34
RCP4.5	2050s	5.10	21.25	67.91	41.98
	2070s	7.44	19.90	64.49	40.15
RCP6.0	2050s	9.66	24.42	63.35	38.95
	2070s	7.23	22.75	63.11	41.86
RCP8.5	2050s	4.66	23.41	68.48	41.97
	2070s	3.21	15.90	66.56	45.94

**Table 3:** Changes in the area of the *L. decastes* complex under future climate models.

Future Climate Scenarios	Period	Newly Acquired Suitable Habitats	Retained Suitable Habitats	Lost Suitable Habitats
RCP2.6	2050s	47.20	26.66	2.03
	2070s	50.21	27.08	1.61
RCP4.5	2050s	49.02	26.87	1.82
	2070s	48.81	26.31	2.38
RCP6.0	2050s	55.80	27.78	0.91
	2070s	49.19	27.81	0.87
RCP8.5	2050s	52.37	27.78	0.91
	2070s	41.54	24.60	4.09

**Table 4:** Changes in the center of mass of the species *L. decastes* complex under different climate modes.

Different Climate Patterns	Period	Longitude	Latitude
Current climate	1985s	100°39′22″	27°37′12″
RCP2.6	2050s	99°52′48″	28°27′35″
	2070s	99°52′48″	28°27′35″
RCP4.5	2050s	99°47′24″	28°35′24″
	2070s	99°49′48″	28°33′18″
RCP6.0	2050s	100°1′12″	28°16′12″
	2070s	100°15′36″	27°58′11″
RCP8.5	2050s	99°57′35″	28°18′36″
	2070s	99°31′48″	29°4′48″

## Data Availability

The original contributions presented in the study are included in the article and Supplementary Materials. Further inquiries can be directed to the corresponding author.

## Supplementary Materials

The following supporting information can be downloaded at: https://www.mdpi.com/article/10.3390/jof12080571/s1, Table S1: Original occurrence records; Table S2: Filtered original occurrence records; Table S3: Collection information and ITS sequence data for specimens collected in the present study; Table S4: Percentage change for the area of *L. decastes* complex in different periods (%).
