# Supplementary material for: Exploration of the Potential Suitable Distribution Range of the Lyophyllum decastes Complex in High-Altitude Cold Regions of Southwest China
Source: J Fungi (Basel). 2026 Aug 1;12(8):571. doi: 10.3390/jof12080571 (PMC13514881; doi:10.3390/jof12080571)
Supplement: Supplementary file 1 [file jof-12-00571-s001.zip › Supplementary Table S4 Percentage change of area of L. decastes complex in different periods (%).pdf]

Table S4. Percentage change of area of *L. decastes* complex in different periods (%)

Different Climate	Period	High Suitable Habitats	Medium-high Suitable Habitats	Medium Suitable Habitats	Low Suitable Habitats
RCP2.6	2050s	-48.23%	-16.96%	6.05%	29.45%
	2070s	-33.19%	-8.95%	3.64%	28.03%
RCP4.5	2050s	-27.66%	-9.46%	2.91%	26.94%
	2070s	5.53%	-15.21%	-2.27%	21.41%
RCP6.0	2050s	37.02%	4.05%	-4.00%	17.78%
	2070s	2.55%	-3.07%	-4.36%	26.58%
RCP8.5	2050s	-33.90%	-0.26%	3.77%	26.91%
	2070s	-54.47%	-32.25%	0.86%	38.92%
